# The relationship of nursing practice laws to suicide and homicide rates: a longitudinal analysis of US states from 2012 to 2016

**DOI:** 10.1186/s12913-020-5025-x

**Published:** 2020-03-06

**Authors:** Kristen R. Choi, Sae Takada, Altaf Saadi, Molly C. Easterlin, Liza S. Buchbinder, Shaw Natsui, Frederick J. Zimmerman

**Affiliations:** 1 National Clinician Scholars Program, Division of General Internal Medicine & Health Services Research, Department of Medicine, David Geffen School of Medicine, University of California, Los Angeles, Los Angeles, CA USA; 2 Department of Health Policy and Management, Fielding School of Public Health, University of California, 1100 Glendon Suite 900, Los Angeles, CA 90024 USA; 3 U.S. Department of Veterans Affairs, Health Services Research & Development, Center for the Study of Healthcare Innovation, Implementation, & Policy, Los Angeles, CA USA; 4 Department of Pediatrics, Cedars-Sinai Medical Center, Los Angeles, CA USA

**Keywords:** Nurse practitioner, Registered nurse, Scope of practice, Injury, Suicide, Policy

## Abstract

**Background:**

Nursing resources can have a protective effect on patient outcomes, but nurses and nursing scope of practice have not been studied in relation to injury outcomes. The purpose of this study was to examine whether scope of practice and ease of practice laws for nurse practitioners and registered nurses are associated with suicide and homicide rates in the United States.

**Methods:**

This state-level analysis used data from 2012 to 2016. The outcome variables were age-adjusted suicide and homicide rates. The predictor variables were NP scope of practice by state (limited, partial, or full) and RN ease of practice (state RN licensure compact membership status). Covariates were state sociodemographic, healthcare, and firearm/firearm policy context variables that have a known relationship with the outcomes.

**Results:**

Full scope of practice for NPs was associated with lower rates of suicide and homicide, with stronger associations for suicide. Likewise, greater ease of practice for RNs was associated with lower suicide and homicide rates.

**Conclusions:**

Findings suggest that nurses are an important component of the healthcare ecosystem as it relates to injury outcomes. Laws supporting full nursing practice may have a protective effect on population health in the area of injuries and future studies should explore this relationship further.

## Background

Injury-related mortality from suicide and homicide is a significant public health issue in the United States (US). Suicide rates have been consistently rising from 1999 to 2016, with average increases of 30% in half of US states since 1999 [1]. Homicide rates, on the other hand, have been decreasing consistently over recent decades with current rates at historic lows [2]. However, there were small increases in homicide rate from 2014 to 2016 [2]. Concerningly, there are also persistent, high racial disparities in homicide rate. The homicide rate for Black/African American populations is 8 times higher than the rate for White populations, while the rate for Hispanic populations is 4 times higher than that of White populations [3].

Firearms play a significant role in both suicides and homicides, and there have been several recent studies on firearm policy and other sociodemographic factors associated with firearm-related and overall injury mortality [4–7]. While these studies have identified the role of some important social and policy factors in states that can affect injury mortality, a key factor that has been understudied is the role of the healthcare system [8–14]. Healthcare resources play a critical role in injury outcomes in two ways: (1) prevention of violence against self or others (e.g., mental health services, psychiatric emergency care, wellness promotion and risk assessment) and (2) post-injury care that saves lives and prevents immediate mortality (e.g., trauma and emergency services, inpatient care, follow-up and rehabilitative care) [15, 16]. A number of healthcare system factors including insurance coverage, availability and quality of healthcare facilities, and availability of appropriate providers, may exert a significant effect on the injury continuum from prevention to treatment [17–19].

Registered nurses (RNs) and nurse practitioners (NPs) are an increasingly important part of the healthcare ecosystem in the US [20]. In inpatient settings, the amount and quality of nursing care patients receive can determine mortality and other health outcomes [21–24]. In outpatient settings, NPs play a growing role in improving overall access to healthcare by practicing as physician extenders or independent providers [25]. Given existing evidence on the role of nurses in healthcare and their effect on health outcomes, we hypothesized that amount of nursing resources in states—as determined by nursing practice laws—would have an association with injury outcomes.

Nursing practice laws are state-level policies that determine the scope, ease, and conditions of practice for RNs and advanced practice nurses (APRNs), including NPs. Scope-of-practice laws for NPs are a contentious policy issue in the US, with some states allowing independent practice for NPs without physician oversight and others requiring physician oversight for all APRN practice [26]. There is substantial evidence that increasing NP scope of practice is not only safe and effective, but also increases access to care for underserved populations [27–29]. For RNs, the key law affecting their practice is the Nurse Licensure Compact. The compact allows RNs to practice in all states that are members of the compact under the same RN license, creating greater ease of practice and mobility [30]. While RN licensure compact laws were originally adopted to reduce regulatory barriers to cross-state nursing practice for registered nurses (i.e., policy to influence ease—but not scope—of practice), NP scope of practice laws have a much longer history of altering and expanding the role of nurses in healthcare (i.e., policy to influence scope of practice) [30–32]. The difference between ease of practice laws and scope of practice laws, while subtle, has led to a different context for passage of policies affecting NPs versus RNs. Currently, these two types of nursing practice laws are passed at the state level independently of one another. By affecting the conditions, autonomy, and ease under which nurses practice, nursing practice laws affect the overall healthcare resources in states, which, by extension, could affect public health outcomes including injury [33]. The purpose of this study was to examine associations between nursing practice laws for NPs and RNs and suicide/homicide rates in the US between 2012 and 2016. Our analysis was exploratory, building on recent state-level analyses of these outcomes by exploring the effect of nursing resources while accounting for the sociodemographic and firearm policy context.

## Methods

### Design

This study used state-level data to conduct a five-year longitudinal analysis from 2012 to 2016. Because our analysis relied on variables that were affected by key provisions and court rulings related to the Patient Protection and Affordable Care Act (ACA), we selected an ACA-implementation to post-implementation timeframe [34]. Our study used the Centers for Disease Control Social-Ecological Framework for Violence Prevention to explore state-level effects of community (sociodemographics), institutional (healthcare system factors), and policy (firearms and firearm policy) factors on our outcomes [35]. The healthcare systems component of our conceptual model is complex and multifactorial, encompassing providers, facilities, access to healthcare, and health policy. Healthcare system resources are an important determinant of health outcomes and as such, we hypothesized that expanded overall healthcare resources via nursing practice laws while adjusting for all other above factors—including multiple healthcare system factors—would result in fewer suicides and homicides at the state level. The study was determined to be exempt from Institutional Review Board regulation at the University of California, Los Angeles because it used publicly available, state-level data with no personal identifiers and was not considered to be human subjects research.

### Data sources and variables

#### Outcome variables

The outcome variables for this analysis were suicide rate and homicide rate. Data were obtained from the Centers for Disease Control Web-based Injury Statistics Query and Reporting System (WISQARS) [36]. We extracted total age-adjusted fatality rates for all 50 states over the five-year study period.

#### Predictor variables

The main predictor variables were nurse practitioner scope of practice laws (limited, partial, full) and RN ease of practice (nursing licensure compact member or non-member). These nursing practice policy variables represent the key healthcare system factor variables of interest under our conceptual model. These variables were extracted from the Cato Institute Freedom in the 50 States Project, which ranks US states by policies that may affect personal and economic freedom [37]. The NP scope of practice variable categorized states on whether NPs are permitted independent practice authority (full), partial practice authority with some prescriptive authority (partial), and limited practice authority with physician oversight required for NP care provision (limited). Higher values on this 3-point ordinal variable denote wider NP scope of practice. The Nurse Licensure Compact status variable denotes membership or non-membership in the compact permitting RNs to practice in multiple states.

#### Covariates

The covariates for this analysis were state sociodemographic characteristics, healthcare system factors, and firearm factors that could influence the relationship between our predictor and outcome variables.

The sociodemographic variables were selected based on prior research on factors associated with our outcomes, including degree of urbanity/rurality and poverty rate. We also examined generosity of Medicaid benefits and worker protection laws as indicators of robustness of state social safety net. Generosity of Medicaid benefits was measured as percentage of the Federal Poverty Line (FPL) that qualified one for Medicaid benefits based on income in each state [38]. The worker index variable was derived from the Cato Institute Freedom in the 50 States Project [37]. We used variables for whether or not a state’s minimum wage exceeded the federal minimum wage, the presence of a short-term disability insurance program, the presence of a right-to-work law (conceptualized for this analysis as non-protective of workers [39]), and a mean cutoff for the worker compensation mandated coverage index. Our worker protections index was the sum of these four items, with higher scores indicating more worker protections. Because the variables in our analysis are highly influenced by urbanity/rurality, we also included two indicators of degree of urbanity/rurality. The first was average county population density per 1000 population. This variable was derived by summing for all counties in a given state, for each year: (county population / county land area) * (county population / state population). The second was a 3-point ordinal variable classifying states as rural, suburban, or urban, with higher values denoting a greater degree of urbanity. This categorization was derived based on share of the nonelderly population residing in a rural area in a given state [40]. Data for this variable were only available for a single year (2015), so the urban/rural classification did not vary by study year.

The healthcare system variables were rates of healthcare providers (primary care physicians, psychiatrists, NPs, RNs) and percentage of the state population residing within a 1-h drive of a Level I or Level II trauma center. We included these items as covariates to adjust for healthcare system factors that might affect the relationship between our main predictors and outcomes. The healthcare provider rate variables (primary care physicians [general and family medicine physicians], psychiatrists, NPs, RNs)—operationalizing physician and nursing resources, respectively—were constructed as number of providers per 1000 persons in each state using counts of employed providers in each category from the Bureau of Labor Statistics [41]. These two physician types were selected because it is within the scope of practice for both providers to assess for, and address, firearm-related risk. The distance to trauma center variable was a 6-point variable derived from a report from the American College of Emergency Physicians in 2014 [42]. States were classified based on the population share residing close to a trauma center, with higher values denoting a larger population share living near a trauma center. We used a distance to trauma center variable because evidence suggests that geographic distance to a trauma center is a important predictor of survival from gunshot wounds, where survival depends on timely care [43, 44]. Those who live far distances from trauma centers or in rural areas with few hospitals (sometimes called “trauma deserts”) may be disadvantaged for receiving care after a gunshot wound such that their likelihood of survival is impacted [43–45]. Data for this variable were only available for a single year (2014), so the distance to trauma center variable did not vary by study year.

For firearm variables, we used a count of firearm-related laws that have a theoretical relationship to the study outcomes and hunting license rate as a partial proxy for gun ownership rate. The State Firearm Laws Database compiles data on state firearm policy from 1991 to 2016 on 133 firearm laws in 14 categories [46]. Our analysis used count of laws by state for two categories that were conceptually linked to our outcomes: prohibitions for high-risk gun possession related to mental illness or substance abuse (firearm possession prohibition for involuntary commitment to a mental health facility drug-related misdemeanor conviction, alcohol-related reasons, or designation by a court of an individual as a danger to self or others), and prohibitions for high-risk gun possession related to history of crime (firearm possession prohibition for felony convictions or violent misdemeanors). There were 6 possible mental health−/substance abuse-related laws and 4 possible violence-related laws. The hunting license rate variable is part of a proxy measure of gun ownership that incorporates both hunting license rate and firearm-related suicide rate [47]. Since our outcome variable included firearm-related suicide rate, we used hunting license rate as a partial proxy that likely captures safe and legal gun ownership.

### Analysis

We used fixed effects linear regression models to explore relationships between nursing practice laws and suicide/homicide rates, with separate models for NP and RN laws and while adjusting for all sociodemographic, healthcare, and firearm covariates. All models included fixed effects for year and US Census Division to account for secular trends and regional clustering in outcomes. After constructing analytic variables, we used frequencies, descriptive statistics, and heat maps to examine study variables and visualize variability among states. Our models examined associations between the nursing practice laws and suicide/homicide, accounting for all other covariates.

For states that were missing homicide outcomes data for certain years (Hawaii, North Dakota, South Dakota, Maine, New Hampshire, Wyoming), we imputed the homicide rate using murder rate as a proxy. For states that were missing data on medical providers for certain years, we imputed missing provider counts by taking the mean of the preceding and following year. Two states, Idaho and South Dakota, were missing data on number of psychiatrists for the majority of the study period and the missing years were omitted from the analysis. Louisiana and Wyoming were missing data on number of psychiatrists in 2012, and as there was no preceding year for these data points to impute they were also omitted. All other data were complete for the study period (*N* = 242).

## Results

**Fig. 1:**
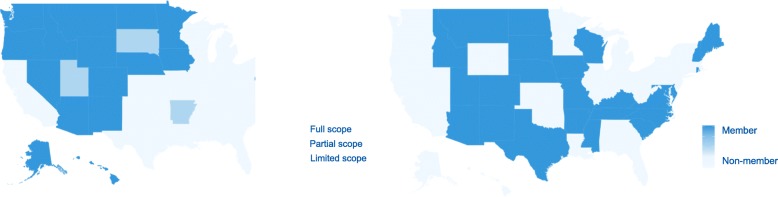
Nursing Practice Laws in US States, 2014. Footnote: Authors’ own work; analysis of data from the Cato Institute Freedom in the 50 States Project, 2012–2016. Exhibit 1a shows differences in nurse practitioner scope of practice laws across the US states in 2014, the median study year (range 1–3). Exhibit 1b shows differences Nurse Licensure Compact membership across the US states in 2014 (Member, Non-member

Over the study period, 54.8% of states were categorized as having limited NP scope of practice laws, 8.0% of states were categorized as partial, and 37.2% of states were categorized as full. Forty-nine percent of states were part of the RN licensure compact agreement (Fig. 1). The average suicide rate was 15.28 deaths per 100,000 population (minimum: 7.14, New Jersey; maximum: 29.78, Wyoming). The average homicide rate was 5.36 deaths per 100,000 population (minimum: 1.69, Utah; maximum: 14.20, Louisiana). States had decreased poverty rates and hunting license rates from 2012 to 2016 and increased Medicaid generosity, density, worker projections, and firearm policies. NP and RN rates increased from 2012 to 2016, bu. tthere were minimal changes to physician rates (see Table 1).

**Table 1:** Analytic Variables

Variable	2012 (Baseline)	2016 (End)
	M (SD)	M (SD)
Poverty rate (%)	18.75(4.82)	16.44(4.31)
Medicaid generosity (% of FPL)	86.68(62.95)	102.50(49.59)
Hunting license rate (licenses per 1000 population)	203(213.2)	191.3(194.6)
Density (per 1000 county population)	1094(1376)	1135(1422)
Primary care physician rate (per1000 population)	0.41(0.19)	0.42(0.21)
Psychiatrist rate (per 1000 population)	0.08(0.03)	0.08(0.06)
NP rate (per 1000 population)	0.38(0.16)	0.51(0.18)
RN rate (per 1000 population)	8.81(1.79)	9.31(1.85)
Worker protections index (0–4)	1.68(1.36)	1.82(1.50)
Firearm policy count: Mental illness/substance abuse	1.30(1.41)	1.44(1.45)
Firearm policy count: Violent offenders	1.20(0.99)	1.26(1.09)

Authors’ analysis of data from the US Census Bureau, Kaiser Family Foundation, US Fish and Wildlife Service, Bureau of Labor Statistics, Cato Institute Freedom in the 50 States Project, and State Firearm Laws Database, 2012–2016. *FPL* federal poverty line, *M* mean, *SD* standard deviation, *RN* registered nurse, *NP* nurse practitioner

In the first set of models (Table 2), full NP scope of practice was associated with fewer suicides and fewer homicides. The association was stronger for suicide than homicide and the presence of for full scope of practice laws. There was also a significant association between higher NP rates and lower homicide rates. Higher population density and state urbanity were associated with fewer suicides, while a higher hunting license rate was associated with more suicides. Poverty rate was positively associated with homicide rate. Unexpectedly, we found a positive relationship between primary care physician rate and both outcome variables and between partial NP scope of practice and suicide. There was also unexpectedly a positive relationship between worker protections and homicide rate, as well as a weak, but significant, negative relationship between hunting license rate and homicide rate.

**Table 2:** Nurse Practitioner Scope of Practice and Suicide/Homicide

	Suicide (R^2^ = 0.84)	Homicide (R^2^ = 0.60)
	β (SE)	β (SE)
Urbanity/Rurality (reference: rural)		
Suburban	− 0.70(0.57)	−1.16(0.53)^a^
Urban	−2.48(0.81)^b^	−1.61(0.76)^a^
Poverty rate	−0.02(0.04)	0.16(0.04)^b^
Hunting license rate	< 0.01(< 0.01)^b^	- < 0.01(< 0.01)^b^
Density	- < 0.01(<.01)^b^	- < 0.01(<.01)
Worker index	−0.37(0.18)	0.30(0.15)^b^
Primary care physician rate	2.84(0.68)^b^	1.12(0.65)
Psychiatrist rate	−1.84(3.61)	−4.02(3.61)
NP rate	−2.11(1.20)	−2.28(1.08)^a^
Medicaid generosity	−0.18(0.32)	−0.04(0.29)
Distance to trauma center	0.21(0.18)	−0.09(0.17)
NP scope of practice (reference: limited)		
Partial scope of practice	1.18(0.54)^a^	−1.51(0.43)^b^
Full scope of practice	−2.32(0.46)^b^	−1.31(0.39)^b^
Firearm prohibition laws	0.25(0.13)	−0.21(0.14)

Authors’ analysis of data from the US Census Bureau, Kaiser Family Foundation, US Fish and Wildlife Service, Bureau of Labor Statistics, Cato Institute Freedom in the 50 States Project, and State Firearm Laws Database. *SE* standard error, *NP* nurse practitioner; ^a^Value is significant at the 0.05 level; ^b^Value is significant at the 0.01 level. This table displays state-level fixed effects linear regression models of nurse practitioner scope of practice laws, sociodemographic and healthcare system factors, firearm policy, and their relationship to suicide and homicide rates for all 50 states, excluding the District of Columbia and US territories. Estimates are adjusted for year and census division. The firearm prohibition laws differ by outcome; for the suicide model, the firearm prohibition laws variable is a count of laws prohibiting firearm possession for those with certain types of mental illness. For the homicide model, the variable is a count of laws prohibiting firearm possession for those with a history of certain types of violent crime

The second set of models (Table 3) examined RN licensure compact status and its relationship to suicide and homicide rates. A very similar pattern of significance was found in the RN models as was found in the NP models. RN licensure compact membership was associated with lower suicide and homicide rates. The direction and significance of covariates for both suicide and homicide was similar to that of the NP models, including the unexpected findings. One difference was that psychiatrist rate was associated with lower homicide rates in the RN models.

**Table 3:** Registered Nurse Ease of Practice and Suicide/Homicide

	Suicide (R^2^ = 0.81)	Homicide (R^2^ = 0.58)
	β (SE)	β (SE)
Urbanity/Rurality (reference: rural)		
Suburban	−0.21(0.59)	−1.16(0.53)^a^
Urban	−1.60(0.77)^a^	−1.05(0.67)
Poverty rate	0.05(0.04)	0.20(0.03)^b^
Hunting license rate	< 0.01(< 0.01)^b^	- < 0.01(<.01)^a^
Density	- < 0.01(<.01)^b^	- < 0.01(<.01)
Worker index	−0.24(0.22)	0.40(0.18)^a^
Primary care physician rate	3.60(0.78)^b^	1.58(0.67)^a^
Psychiatrist rate	−5.55(3.82)	−7.25(3.48)^a^
RN rate	0.06(0.15)	−0.19(0.13)
Medicaid generosity	0.08(0.35)	−0.16(0.30)
Distance to trauma center	0.04(0.18)	< 0.01(0.16)
RN ease of practice	−0.60(0.30)^a^	−0.67(0.27)^a^
Firearm prohibition laws	−0.15(0.13)	−0.27(0.14)

Authors’ analysis of data from the US Census Bureau, Kaiser Family Foundation, US Fish and Wildlife Service, Bureau of Labor Statistics, Cato Institute Freedom in the 50 States Project, and State Firearm Laws Database. *SE* standard error, *RN* registered nurse; ^a^Value is significant at the 0.05 level; ^b^Value is significant at the 0.01 level. This table displays state-level fixed effects linear regression models of RN ease of practice laws, sociodemographic and healthcare system factors, firearm policy, and their relationship to suicide and homicide rates for all 50 states, excluding the District of Columbia and US territories. Estimates are adjusted for year and census division. The firearm prohibition laws differ by outcome; for the suicide model, the firearm prohibition laws variable is a count of laws prohibiting firearm possession for those with certain types of mental illness. For the homicide model, the variable is a count of laws prohibiting firearm possession for those with a history of certain types of violent crime

## Discussion

This study found associations between full scope of practice for NPs and lower rates of suicide and homicide at the state level in the US. Likewise, greater ease of practice for RNs was associated with lower suicide and homicide rates. These findings suggest that nurses are an important component of the healthcare ecosystem as it relates to injury outcomes, and as such, law favorable to full nursing practice have a protective relationship to population health including injuries. At the population level, nursing practice laws may improve the healthcare ecosystem available for the entire spectrum of injury sequelae, from health promotion and risk reduction of violence against self or others to emergency injury treatment and follow-up care that prevents injury mortality. Prior studies of nursing practice laws have found an effect on healthcare resources and patient outcomes as they relate to inpatient hospitalization and medical care [27, 28]. Our study extends these findings by linking nursing practice laws to injury outcomes and suggesting that nurses are beneficial to population health outcomes more broadly than has been identified previously.

Although there were similarities in findings across models and type of nursing practice laws, the mechanisms by which nursing practice laws may affect suicide and homicide outcomes may be different—and also different by practice laws affecting NPs versus RNs. Suicide occurs more frequently in rural, low-density geographic areas, and there is a very strong known relationship between firearm ownership and suicide [4]. Our analysis supports these findings, with urbanity/density negatively associated with suicide and hunting license rate as a partial proxy for gun ownership positively associated with suicide. There is evidence that nurse NPs more likely to practice in rural areas than other types of providers, and it is possible that expanded NP scope of practice incentivizes practice in rural areas and leads to increased resources for mental health in primary care, psychiatry, community health, and other settings [48, 49]. This pathway would explain our findings between full NP scope of practice and lower suicide rates. A similar phenomenon may be occurring for RNs. When RNs are able to more easily practice in multiple states, the nursing workforce may have opportunity to grow in rural states that are compact members and increase healthcare resources available to the population [33]. RNs are more likely to affect suicide outcomes via services provided in inpatient settings that tend to be more intensive or for acute conditions (e.g., inpatient psychiatric treatment, emergency care), but also practice in community health settings, home visits, and outpatient clinics where they may provide preventive mental health services or assessment and triage that engage patients in services and prevent suicide attempts. This analysis was associative and ecological and as such cannot confirm these mechanisms; however, future studies should identify more specifically mechanisms and pathways by which nursing practice laws affect suicide. Mediation analyses may also be warranted at a more granular level of analysis.

Homicide in the US occurs at higher rates in higher-density southeastern and midwestern states, and nurses may be less able to intervene directly to prevent homicides in the way they can with suicides in clinical care. At a population level, homicides are associated with poverty, social inequality, unemployment, racial segregation, and other forms of socioeconomic resource deprivation [9, 13–15]. Victims of homicides are disproportionately young adults, male, residents of urban areas, and racial/ethnic minorities [2, 50]. Almost half of homicide victims are known to the perpetrator, and most homicides (7 in 10) are perpetrated with a firearm [50]. Given these patterns in homicide, we suspect that nursing practice laws decrease homicide rate by improving healthcare resources available to victims after a homicide attempt and increasing the likelihood of survival (emergency care, trauma care, follow-up and long-term care) rather than by improving healthcare resources such that attempts are prevented, as may be occurring with suicide. Our analysis found a positive relationship between poverty and homicide, which support existing literature on this relationship. These mechanisms cannot be confirmed with the current analysis but should be explored in future research studies.

Unexpectedly, we found a positive relationship between primary care physician rate and suicide/homicide rate across all models. There is limited research on this topic; however, prior studies in countries outside the US have identified a positive relationship between physician density and suicide, for both psychiatrists and physicians in general [51, 52]. These studies posited that areas with higher baseline suicide levels could attract more policy attention and subsequent allocation of physician resources; that primary care practice generally does not allow general practitioners to assess or address suicide; and that there is more accurate reporting of suicide from higher-income localities, which also have more physician resources and thus may confound the relationship. There is no evidence or conceptual reason we can identify that physician rate should have a positive causal relationship with injury outcomes, and it is possible that this finding is related to omitted variable bias for one or more of the reasons discussed in other studies. The role of physician rate and density should be explored in future studies, ideally at a more granular level of analysis than the state level. We also found a positive relationship in one model between partial NP scope of practice and suicide. The partial scope of practice variable is defined by expanded prescriptive authority—but not independent practice authority—for NPs. Evidence suggests that NPs prescribing patterns for medications including opiates are comparable to that of physicians [53], and the relationship between prescriptive authority and patient outcomes requires further study as there are few studies comparing full versus partial scope of practice for NPs.

There was also a positive relationship between worker protection laws and homicide and a negative relationship between hunting license rate and homicide. These findings are contradictory and unexpected, as prior studies have identified positive associations between gun ownership and homicide and unemployment and homicide [54–56]. Possible explanations for these findings are that our gun ownership proxy variable, hunting license rate, may partially capture rurality and drive the weak, negative association found in our analysis; and that omitted variable bias drives the positive relationship between worker protections and homicides. We recommend further studies of these variables at a more granular level of analysis to confirm or refute these findings and advise caution around their interpretation as they contradict established conceptual models for understanding injury-related mortality.

This study has several strengths and limitations that should be considered. We used recent, longitudinal data to explore relationships between nursing practice laws and injury outcomes with substantial controls for the sociodemographic and policy context. This includes both healthcare system variables and firearm-related variables that have not traditionally been studied together. The limitations are the ecological nature of this analysis which prevents inferences at the individual level, an inability to identify mechanisms driving associations found in this analysis, and a lack of more granular data. The gun ownership variable was a proxy measure which may not have ideally captured actual gun ownership at the state level. The distance to trauma center and urbanity/rurality variables were only available for a single year and thus did not vary across states by year. It is possible that we did not capture recent trends in urbanization and closure of rural hospitals and may have under-estimated the magnitude of association for these variables [45]. A small number of states did not have homicide data available, and our proxy measure only partially captures homicide. Given our contradictory findings around physician rates and homicide, some of the measures used to operationalize healthcare system factors may not have ideally captured the true constructs of interest, or these relationships may suffer from omitted variables bias. It is possible that there are other omitted variables our analysis did not account for that may affect the relationships under study. It is also important to note that although we observed significant relationships at the state level between nursing practice laws and injury outcomes, nursing and overall healthcare system factors may play a relatively minor role in the overall landscape of social, political, and other determinants of injury outcomes.

## Conclusions

Nurses are an essential healthcare profession in the US and play a key role in population health outcomes. As such, laws affecting their practice may indirectly affect population health outcomes. This study found that laws allowing full scope of practice for NPs and greater ease of practice for RNs were both associated with lower population-level rates of suicide and homicide, though this relationship was not observed for partial NP scope of practice laws. Future studies should explore the mechanisms by which nursing practice laws affect injury outcomes so that such laws can be considered as part of a strategy to improve public health and reduce injury-related mortality.

## Data Availability

The data used in the current study are from a variety of publicly available sources: the Centers for Disease Control Web-based Injury Statistics Query and Reporting System (WISQARS); the Cato Institute Freedom in the 50 States Project; the Kaiser Family Foundation; the US Census Bureau; the Bureau of Labor Statistics; the American College of Emergency Physicians; the State Firearm Laws Database; and the US Fish and Wildlife Service.

## Abbreviations

ACA: Affordable Care Act
APRN: Advanced practice registered nurse
CDC: Centers for Disease Control and Prevention
FPL: Federal poverty line
NP: Nurse practitioner
RN: Registered nurse
SE: Standard error
US: United States
WISQARS: Web-based Injury Statistics Query and Reporting System
